# Entropy Production in a System of Janus Particles

**DOI:** 10.3390/e27020112

**Published:** 2025-01-23

**Authors:** Andrés Arango-Restrepo, Juan David Torrenegra-Rico, J. Miguel Rubi

**Affiliations:** Condensed Matter Department, Universitat de Barcelona, 08028 Barcelona, Spain; jdtorrenegrar@unal.edu.co (J.D.T.-R.); mrubi@ub.edu (J.M.R.)

**Keywords:** active particles, energy dissipation, entropy production, janus particles, self-assembly

## Abstract

Entropy production is a key descriptor of out-of-equilibrium behavior in active matter systems, providing insights into both single-particle dynamics and emergent collective phenomena. It helps determine transport coefficients and phoretic velocities and serves as a crucial tool for understanding collective phenomena such as structural transitions, regime shifts, clustering, and self-organization. This study investigates the role of entropy production for individual active (catalytic Janus) particles and in systems of active particles interacting with one another and their environment. We employ a multiscale framework to bridge microscopic particle dynamics and macroscopic behavior, offering a thermodynamic perspective on active matter. These findings enhance our understanding of the fundamental principles governing active particle systems and create new opportunities for addressing unresolved questions in non-equilibrium thermodynamics.

## 1. Introduction

Active particles are small entities that can convert the chemical energy of their environment into mechanical motion. Unlike passive particles, which eventually settle into a stable, equilibrium state (called thermodynamic equilibrium), active particles consume energy continuously, keeping them in a constant state of activity. This continuous consumption of energy leads to unique and complex behaviors that are very different from those observed in systems at equilibrium. Examples of active particles include synthetic micromotors, Janus colloids (particles designed with two distinct sides), and biological entities such as bacteria and cells. These systems are studied in various fields because they provide information on how self-organization, transport, and energy conversion occur in both living and artificial systems [1,2,3,4,5,6,7,8,9].

Thermodynamic principles are essential to understand the behavior of active particles, which interact with their environment by continuously consuming energy and forming out-of-equilibrium structures [10,11]. In systems in equilibrium, energy flows are balanced and entropy production vanishes. In contrast, active particles maintain motion by consuming energy locally, maintaining stable out-of-equilibrium states characterized by persistent dissipation and entropy production [1,12]. For the purposes of this study, we emphasize that the presence of surface gradients, which lead to gradients in surface tension, is the central mechanism driving the system’s behavior. These gradients, arising from propulsion mechanisms such as chemical reactions, light absorption, or thermal effects, create effective potentials [13,14] and induce local fluxes in the surrounding medium [15,16]. This dynamic interplay between surface tension gradients, motion, and dissipation highlights the necessity of extending classical thermodynamic frameworks to accurately describe systems far from equilibrium [17].

To rigorously study these behaviors, researchers employ advanced theoretical and experimental tools. Fluctuation theorems play a critical role by describing the statistical properties of entropy production and work in systems driven far from equilibrium [18,19], while stochastic thermodynamics models energy exchanges on the scale of individual particles, tracking heat, work, and entropy along their trajectories [20,21,22,23]. For collective behaviors, mesoscopic non-equilibrium thermodynamics (MNET) has emerged as a powerful approach, capturing nonlinearities through Fokker–Planck type kinetic equations that describe the evolution of mesoscopic variables and their associated entropy production [24]. MNET provides a robust framework for studying out-of-equilibrium systems and acts as a bridge between microscopic dynamics and macroscopic behaviors. It extends classical thermodynamics by incorporating mesoscopic variables, allowing the analysis of systems with inherent fluctuations and complex, nonlinear interactions [25]. In particular, MNET captures nonlinear relationships between fluxes and forces, leading to the law of mass action in chemical reactions [26], where the driving force is not affinity but a difference in fugacity [27].

These methodologies provide a framework for analyzing the connection between individual and collective dynamics and fundamental thermodynamic processes. However, our approach emphasizes mesoscopic entropy production rather than stochastic thermodynamics, as our goal is to describe mesoscale collective behavior while linking entropy production to dynamics. Specifically, we aim to (1) use entropy production to characterize different regimes, (2) apply interfacial entropy production to estimate transport coefficients, and (3) investigate entropy production at the particle surface due to chemical reactions while accounting for entropy production in the bulk. Our study explores the utility of entropy production in active particle systems, focusing on catalytic Janus particles. Rather than simply calculating entropy production, we aim to demonstrate its potential as a powerful descriptor, positioning it not merely as a non-equilibrium quantity but also as a fundamental variable for understanding and describing active matter systems.

This paper presents a detailed investigation of the theories and methodologies for analyzing active particles, focusing on the calculation and application of entropy production. By exploring both the dynamics of individual particles and systems of active particles interacting with the medium and each other, we highlight entropy production as a central thermodynamic quantity for understanding non-equilibrium processes in active particle dynamics. Our study examines how entropy production at the mesoscale links microscopic particle behavior with emergent macroscopic phenomena, such as clustering and self-organization. This approach provides new insights into the thermodynamic principles governing active systems, which advances our understanding of non-equilibrium dynamics and offers potential applications in the design of functional materials and the study of biological processes at all scales.

## 2. Active Particles Dynamics

Entropy production and particle dynamics are intrinsically interconnected. Historically, however, dynamics and thermodynamics have often been treated as distinct disciplines, and the field of active matter is no exception. Despite this separation, models describing the dynamics of active particles are fundamental to understanding the non-equilibrium nature of these systems. In this section, we present a range of models that capture the dynamics of active particles, spanning from the molecular to the macroscopic scale.

**Figure 1:**
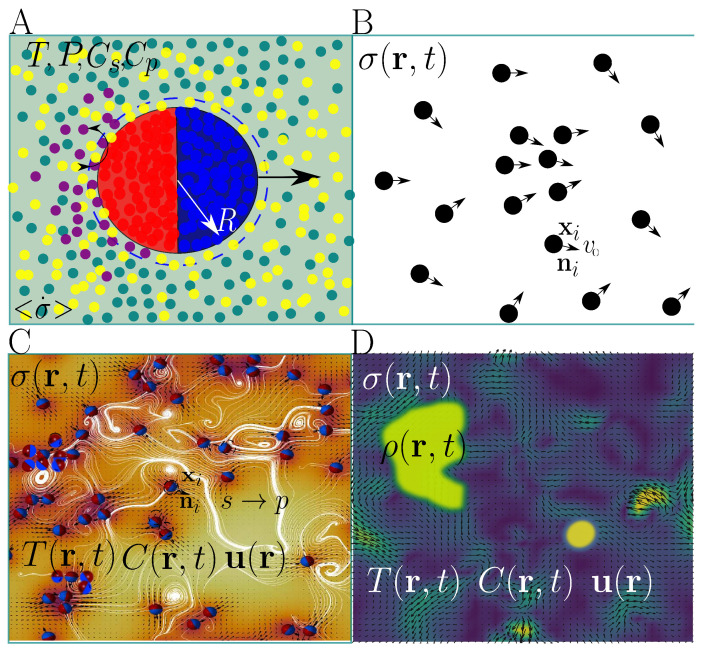
Models for active particle dynamics and thermodynamics. (**A**) Dissipative molecular dynamics: Focuses on the dynamics of individual particles, capturing interfacial concentration gradients, *C*, and temperature, *T*, near the particle surface. The entropy production at this scale fluctuates; the time-averaged entropy production, 〈σ〉, is calculated under open, stable, and thermostated conditions. (**B**) Langevin/Vicsek models. Mainly used to track particle positions, $r_{i}$, and orientations, $n_{i}$, with a fixed active velocity, $v_{0}$. The entropy production is calculated as a time-averaged quantity, ${\langle \sigma \rangle }_{t}$, over the particle trajectories. (**C**) Multiscale models: Combine the effects of concentration fields, C(r,t), temperature, T(r,t), and velocity, u(r,t), on the dynamics and thermodynamics of active particles. They integrate the continuous nature of thermodynamic fields with the discrete nature of particles, enabling the calculation of the local entropy production, σ(r,t), using mesoscopic approaches. (**D**) Macroscopic/continuous models: Assume a continuous particle distribution, ρ(r,t). In this framework, entropy production, σ(r,t), is derived using mesoscopic or linear irreversible thermodynamic approach.

Figure 1 illustrates the system and key variables for various modeling approaches, including dissipative molecular dynamics, Langevin/Viscek models, multiscale frameworks, and macroscopic continuum models. Dissipative molecular dynamics focuses on describing the dynamics of individual particles, capturing interfacial processes and gradients near the particle surface [28,29]. Entropy production inherently fluctuates on this scale, and its time average is calculated under thermostated steady-state conditions in open systems [30]. Langevin models are designed to study the emergence of collective behavior, accounting for the positions and orientations of active particles [7,9,31]. In these models, entropy production is typically calculated as a path-averaged quantity [21]. Multiscale models have recently gained popularity due to their ability to couple the discrete nature of particles with continuous representations of temperature, concentration, and hydrodynamic fields [32,33,34,35]. Given the interplay of multiple scales in describing active particle dynamics and thermodynamic quantities, a mesoscopic approach to entropy production is often the most appropriate [25]. Continuum models simplify numerical and analytical calculations by assuming a continuous particle distribution and using free energy functionals to describe self-assembling process through Ginzburg–Landau dynamics and kinetic theory [36,37]. Although some microscopic details are lost, these models allow analytical studies to be performed [38].

Here we use the MNET formalism to compute entropy production and describe the dynamics, because it unites microscopic and macroscopic descriptions and effectively accounts for nonlinearities and dissipation due to surface gradients, in bulk and interactions [39]. However, combining MNET with elements of stochastic thermodynamics can provide additional information on fluctuations at the individual particle level, especially in systems with very small particles.

Non-equilibrium molecular dynamics simulations are widely employed to explore the microscopic behavior of active particles, offering insight into surface phenomena [28,40,41,42]. In these simulations, the solvent and the active particles are typically represented with Lennard-Jones particles that interact according to the potential

(1) $$u_{ij}=\left[4\varepsilon_{ij}\left({\left(\frac{a}{r}\right)}^{12}-{\left(\frac{a}{r}\right)}^{6}-u\left(r_{c}\right)\right)\right]\Theta \left(r_{c}-r\right)$$

in which $\varepsilon_{ij}$ is the interaction strength between particles of fluid (*i*) and active particles (*j*), *a* is the diameter of the particles, *r* is the distance between particles *i* and *j*, Θ is the Heaviside function, and $r_{c}$ is an interaction cut-off. These simulations enable the direct computation of thermodynamic quantities and transport coefficients for non-interacting active particles, providing insights into entropy production and energy dissipation at the molecular scale.

Langevin dynamics offers stochastic descriptions of the collective motion of particles. Langevin equations account for the interactions among particles, propulsion forces, thermal noise, and dissipation, providing a trajectory-level perspective on work and heat exchange. The typical set of equations is inspired by the Vicsek model [31], which describes the overdamped Langevin dynamics for the position $r_{i}$ and orientation $n_{i}$ of the ith-particle in a solution in the presence of a substrate *s* with a concentration $C_{s}$

(2) $${\dot{r}}_{i}=v_{0}n_{i}+\xi_{t}\nabla C_{s}\left(r_{i}\right)+R_{i}\left(t\right)$$

(3) $${\dot{n}}_{i}=\xi_{r}\left(1-n_{i}n_{i}\right)\nabla C_{s}\left(r_{i}\right)+N_{i}\left(t\right)\times n_{i}$$

Here, the random terms arising from thermal fluctuations satisfy the fluctuation–dissipation theorems $\langle R_{i}R_{i}\rangle =2D_{t}I\delta \left(t-t^{\prime }\right)$ and $\langle N_{i}N_{i}\rangle =2D_{r}I\delta \left(t-t^{\prime }\right)$; $v_{0}$ is the active velocity, $\xi_{t}$ is the diffusiophoretic coefficient, $\xi_{s}$ is the phoretic rotation coefficient, $D_{t}$ is the particle diffusivity, and $D_{r}$ is the rotation diffusivity of the particle [7].

The substrate dynamics has been analyzed to account for non-constant active velocities by expressing the self-diffusiophoretic velocity as a function of the reaction rate and substrate concentration [39]. The resulting Langevin dynamics for the particle position and the conservation equation for the substrate are

(4) $${\dot{r}}_{i}=\xi_{s}C_{s}n_{i}+\xi_{t}\nabla C_{s}\left(r_{i}\right)+R_{i}\left(t\right)$$

(5) $$\frac{\partial C_{s}}{\partial t}=D_{s}\nabla^{2}C_{s}-\sum_{i}^{N_{p}}J_{i}\left(r_{i}\right)$$

Here, the diffusiophoretic velocity is $\xi_{s}C_{s}$, with $\xi_{s}$ the self-diffusiophoretic coefficient and $J_{i}\left(r_{i}\right)$ the reaction rate at the particle position. As discussed in Ref. [39], fluctuations in diffusive and reactive fluxes are negligible compared to the implicit noise in $J_{i}$ caused by the thermal fluctuations of $r_{i}$, particularly when the particle radius exceeds 100 nm. Consequently, Equation (5) does not include an explicit noise source term, and the entropy production rate does not explicitly depend on noise but implicitly on fluctuating particle position.

On the experimental side, various techniques have been developed to measure entropy production and energy flows in active systems from active particle dynamics. For example, high-resolution particle tracking allows one to extract trajectory-level data, which facilitates the computation of dissipative forces and energy consumption [43,44]. Calorimetric methods are used to directly measure the heat released during propulsion, which provides information on the efficiency of energy conversion [45]. Other approaches include using tracer particles to study the hydrodynamic and chemical fields generated by active particles, providing indirect measurements of the energy fluxes involved [46]. These tools offer a comprehensive view of the thermodynamics governing active particles, bridging the gap between microscopic processes and macroscopic emergent phenomena. In the next sections, we focus on computing entropy production at both particle and active system levels.

## 3. Single-Particle Entropy Production

**Figure 2:**
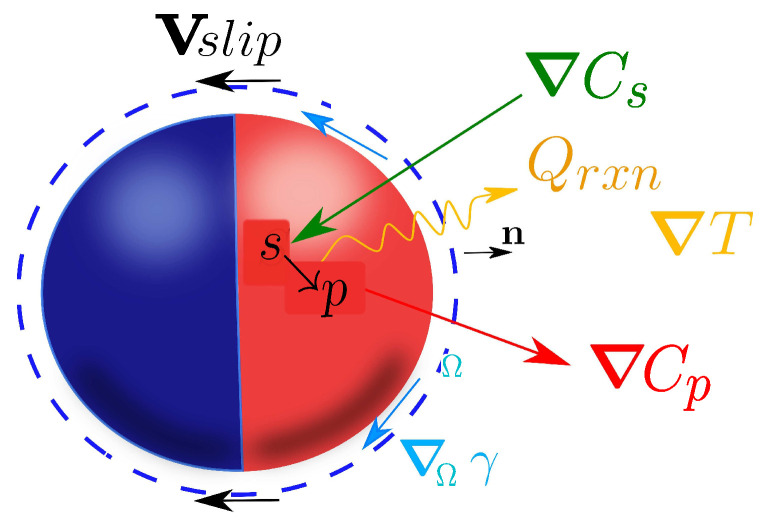
Schematic representation of a catalytic Janus particle, illustrating the reactive interface where the chemical reaction s→p occurs. This reaction generates concentration gradients, $\nabla C_{s}$ and $\nabla C_{p}$, as well as temperature gradients, ∇T, both at the interface and in the surrounding medium. These gradients result in changes in surface tension, $\nabla_{\Omega }\gamma$, which induce the slip velocity, $v_{slip}$.

Entropy production is a key thermodynamic concept for understanding the motion of active particles, particularly in phoretic mechanisms where surface tension gradients at the particle–fluid interface drive propulsion. Figure 2a illustrates a typical schematic representation of a Janus catalytic particle. In this system, a chemical reaction converts substrate *s* to product *p*, releasing heat $Q_{rxn}$. The reaction generates surface gradients in surface tension γ, caused by gradients in concentration ($C_{s}$,$C_{p}$) and temperature (*T*) at the particle–bulk interface. These variations in surface tension induce a slip velocity, $v_{slip}$, that defines the velocity of the active particle.

By using Onsager’s reciprocal relations [47,48], one can calculate slip velocities in the presence of temperature, concentration, or pressure gradients [11,15,16,49,50,51] from entropy production, which is derived by combining mass, energy, and momentum balances with the second law of thermodynamics [26]. These gradients induce radial flows and fluid flows, linking chemical reactions with mechanical motion and providing a framework for analyzing energy dissipation. The entropy production rate at the particle surface is given by

(6) $$\sigma^{\Omega }=-\frac{1}{T}m\cdot P^{\Omega }\cdot \left(1-mm\right)\cdot v_{slip}-\frac{1}{T}J_{s}^{\Omega }\cdot \nabla_{\Omega }\mu_{s}^{\Omega }-\frac{1}{T}J_{p}^{\Omega }\cdot \nabla_{\Omega }\mu_{p}^{\Omega }-\frac{1}{T^{2}}J_{q}^{\Omega }\cdot \nabla_{\Omega }T^{\Omega }$$

in which the dissipative forces are the slip velocity, $v_{slip}$, chemical potential surface gradients of substrate, $\nabla_{\Omega }\mu_{s}^{\Omega }$, and product, $\nabla_{\Omega }\mu_{p}^{\Omega }$, and temperature surface gradient, $\nabla_{\Omega }T^{\Omega }$. From now on, using simplified notation, all gradients and variables will be assumed to be interfacial unless otherwise specified. From Equation (6), we can obtain equations for the interfacial pressure, P; m is the normal vector over the surface, and the equation also includes the surface diffusive fluxes of the substrate, $J_{s}$, and product, $J_{p}$, as well as the surface heat flux, $J_{q}$ [11,50]

(7) $$m\cdot P\cdot \left(1-mm\right)=-\frac{L_{vv}}{T}v_{slip}-\frac{L_{vs}}{T}\nabla \mu_{s}-\frac{L_{vp}}{T}\nabla \mu_{p}-\frac{L_{vq}}{T^{2}}\nabla T$$

(8) $$J_{s}=-\frac{L_{sv}}{T}v_{slip}-\frac{L_{ss}}{T}\nabla \mu_{s}-\frac{L_{sp}}{T}\nabla \mu_{p}-\frac{L_{sq}}{T^{2}}\nabla T$$

(9) $$J_{p}=-\frac{L_{pv}}{T}v_{slip}-\frac{L_{ps}}{T}\nabla \mu_{s}-\frac{L_{pp}}{T}\nabla \mu_{p}-\frac{L_{pq}}{T^{2}}\nabla T$$

(10) $$J_{q}=-\frac{L_{qv}}{T}v_{slip}-\frac{L_{qs}}{T}\nabla \mu_{s}-\frac{L_{qp}}{T}\nabla \mu_{p}-\frac{L_{qq}}{T^{2}}\nabla T$$

in which the coefficients $L_{ij}$ are the Onsager’s matrix elements. From this analysis, and considering the conservation equations for species and energy, the slip velocity can be computed, from which the active particle velocity can be derived [11,50].

Phoretic propulsion mechanisms are based on interfacial asymmetries induced by concentration and temperature gradients. In self-diffusiophoresis, surface reactions generate gradients of reactants and products, resulting in sliding velocities that drive particle motion [52,53,54]. Similarly, in thermophoresis and electrophoresis, temperature and electrochemical gradients induce directed motion. Classical models, such as the Derjaguin–Anderson model [13,14], describe these processes, while recent advances in chemical–mechanical coupling have improved the quantification of transport coefficients, dissipation rates, and energy conversion efficiencies [55].

From Equation (7), the slip velocity at the particle–bulk interface can be determined, providing a detailed description of the interfacial flow. By integrating this slip velocity times the friction coefficient over the particle’s surface, the force over the particle is obtained:

(11) $$\begin{array}{cc} F=\xi \int_{\Omega }v_{slip}d\Omega & =-\xi \int_{\Omega }\frac{T}{L_{vv}}m\cdot P\cdot \left(1-mm\right)d\Omega \\ & \;-\xi \int_{\Omega }\frac{L_{vs}}{L_{vv}}\nabla \mu_{s}d\Omega -\xi \int_{\Omega }\frac{L_{vp}}{L_{vv}}\nabla \mu_{p}d\Omega -\xi \int_{\Omega }\frac{L_{vq}}{TL_{vv}}\nabla Td\Omega \\ & =F_{H}+F_{ph} \end{array}$$

This force is expressed as a combination of contributions from the pressure component (hydrodynamic force $F_{H}$) and the temperature and chemical potential/concentration gradients at the surface (phoretic force $F_{ph}$). This result aligns with the Faxén theorem, which relates the phoretic force on a particle to the integral of the surface gradient of the surface tension [16]. Crucially, this equivalence enables the identification of Onsager coefficients, allowing for the computation of phoretic coefficients that quantitatively connect interfacial processes to the resulting particle motion [56].

Since chemical reactions are the main source of entropy production and directly modify interfacial concentrations and temperatures, they provide key information on the self-phoretic mechanism. This understanding allows not only the calculation of slip velocities but also the determination of self-phoretic coefficients and interfacial transport properties. Assuming symmetry in the azimuthal angle, φ, describing the particle surface, the entropy production at the interface as a function of the polar angle, θ, is [56]

(12) $$\sigma \approx \frac{D_{s}R_{g}T_{0}}{C_{0}TR^{2}}{\left(\frac{\partial C_{s}}{\partial \theta }\right)}^{2}+\frac{D_{s}R_{g}T_{0}}{C_{0}TR^{2}}{\left(\frac{\partial C_{p}}{\partial \theta }\right)}^{2}+\frac{\kappa }{T^{2}R^{2}}{\left(\frac{\partial T}{\partial \theta }\right)}^{2}+\frac{R_{g}T_{0}}{T}k_{r}{\left(\Delta z\right)}^{2}>0$$

The first and second terms represent the entropy production contributions from the diffusion of substrate and product, respectively, both proportional to the square of their concentration gradients. The third term captures the entropy production due to conductive heat transport along the interface, depending on the square of the temperature gradient. Finally, the last term quantifies the entropy production associated with chemical reactions, expressed as the square of the fugacity difference, $\Delta z=e^{\mu_{s}/k_{B}T}-e^{\mu_{p}/k_{b}T}$. This expression uses the formulation of entropy production based on fugacity rather than affinity, providing an alternative framework that extends beyond the classical linear relationship with affinity [27,57]. This approach provides a more general and accurate description of standard chemical reactions. Additionally, the entropy production depends on various thermodynamic properties and transport coefficients, including the universal gas constant, $R_{g}$, the initial substrate concentration, $C_{0}$, substrate diffusivity at the interface, thermal conductivity at the interface, κ, and the reaction rate constant, $k_{r}$.

**Figure 3:**
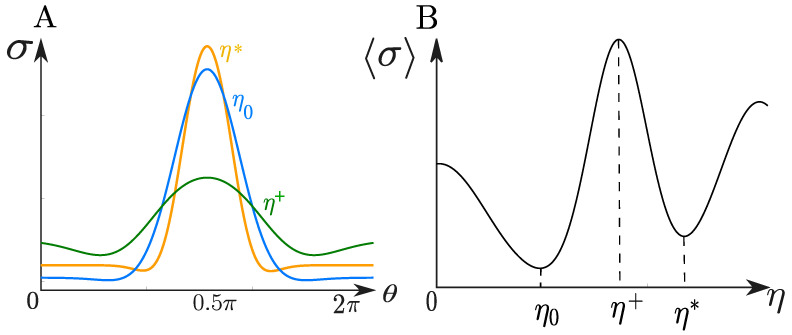
Sketch illustrating the interfacial entropy production for a single catalytic Janus particle. (**A**) Interfacial entropy production rate as a function of the polar angle, θ, describing the particle surface, for different values of the interfacial viscosity. (**B**) Average entropy production rate at the interface as a function of interfacial viscosity, η. Here, $\eta_{0}$ represents the bulk viscosity, $\eta^{†}$ denotes a possible value for the interfacial viscosity, and $\eta^{*}$ corresponds to the actual interfacial viscosity.

Figure 3A illustrates a typical profile of the entropy production rate along the interface, plotted as a function of the orientation angle of the particle, θ. Since entropy production is influenced by various transport coefficients, such as the interfacial viscosity, η (which affects the surface diffusion coefficient $D_{s}$), the figure depicts several entropy profiles corresponding to different values of η. This variation highlights the sensitivity of entropy production to changes in interfacial transport properties, offering insights into how these coefficients influence the thermodynamic behavior of the system.

The average interfacial energy dissipation rate (entropy production rate multiplied by temperature) for a catalytic Janus particle undergoing a first-order exothermic reaction is given by [56]

(13) $$\langle T\sigma \rangle \approx f\left(U,k_{r},D_{s}\right){\langle C_{s}\rangle }^{2}+g\left(U,k_{r},D_{s},U_{q},\Delta H_{r}\right)\langle C_{s}\rangle$$

in which *f* and *g* are functions of the transport coefficients and thermodynamic properties. The energy dissipation was computed by accounting for substrate and product diffusion, heat conduction, and chemical reactions along the interface [56]. It was observed that dissipation scales linearly and quadratically with the average substrate concentration at the interface.

The entropy production depends on the mass transport coefficient, *U*, heat transport coefficient, $U_{q}$, and the interfacial diffusivity, $D_{s}$, quantities that are often challenging to estimate. Notably, the analysis revealed the existence of local maxima in the average energy dissipation as a function of these transport coefficients. This leads to the conclusion that the actual values of these coefficients can be optimized to maximize such non-equilibrium quantities [56] following the maximum entropy production principle [58]. Figure 3B illustrates a schematic profile of the average entropy production at the particle–bulk interface as a function of interfacial viscosity, η. This depiction highlights the potential for estimating the actual value of η by identifying characteristic trends or critical points in the entropy production profile.

## 4. Active Particles Entropy Production During Self-Organization

The thermodynamic framework for collective behavior in active systems advances our understanding of non-equilibrium principles, with implications for material design and engineering. Insights into clustering, phase transitions, and pattern formation can inform the development of self-assembling materials and microfluidic systems, revealing universal principles applicable in physics, biology, and engineering [59,60]. By framing these behaviors within a thermodynamic context, one can uncover universal principles governing non-equilibrium systems, with applications spanning physics, biology, and engineering.

Self-organization in active matter arises from individual particles’ ability to generate local energy flows and respond collectively to interactions, giving rise to phenomena such as swarming, vortex formation, and motility-induced phase separation [17,36,61,62,63,64,65]. On the other hand, traditional tools, such as order parameters, are used to characterize out-of-equilibrium phase transitions. However, these approaches often ignore the irreversible dynamics that drive these transitions and their critical properties. A key bridging concept is entropy production, which has been explored to characterize phase transitions [66,67]. Analyses have been conducted for both continuous and discontinuous phase transitions, extending beyond mean field theories to capture richer nonlocal dynamics, calculating the local entropy production, and providing a more comprehensive understanding of phase behavior in out-of-equilibrium systems [68]. For instance, in clustering and swarming, stochastic thermodynamics have provided tools to quantify entropy production and dissipation [22,69,70,71] to understand the existence of difference regimes.

Mesoscopic non-equilibrium thermodynamics offers a robust framework to address the nonlinearities in active matter, bridging micro- and macro-scales [25,72]. It proposes a probability density, *p*, of finding the reactive system composed of active particles at state Γ and time *t*, which obeys the conservation probability

(14) $$\frac{\partial p}{\partial t}=-\nabla_{\Gamma }\cdot J$$

with J being the probability current in Γ-space. The theory formulates the Gibbs equation, which describes entropy changes in the system:

(15) $$\delta S=-\frac{1}{T}\int \mu \left(\Gamma ,t\right)\delta p\left(\Gamma ,t\right)d\Gamma$$

**Figure 4:**
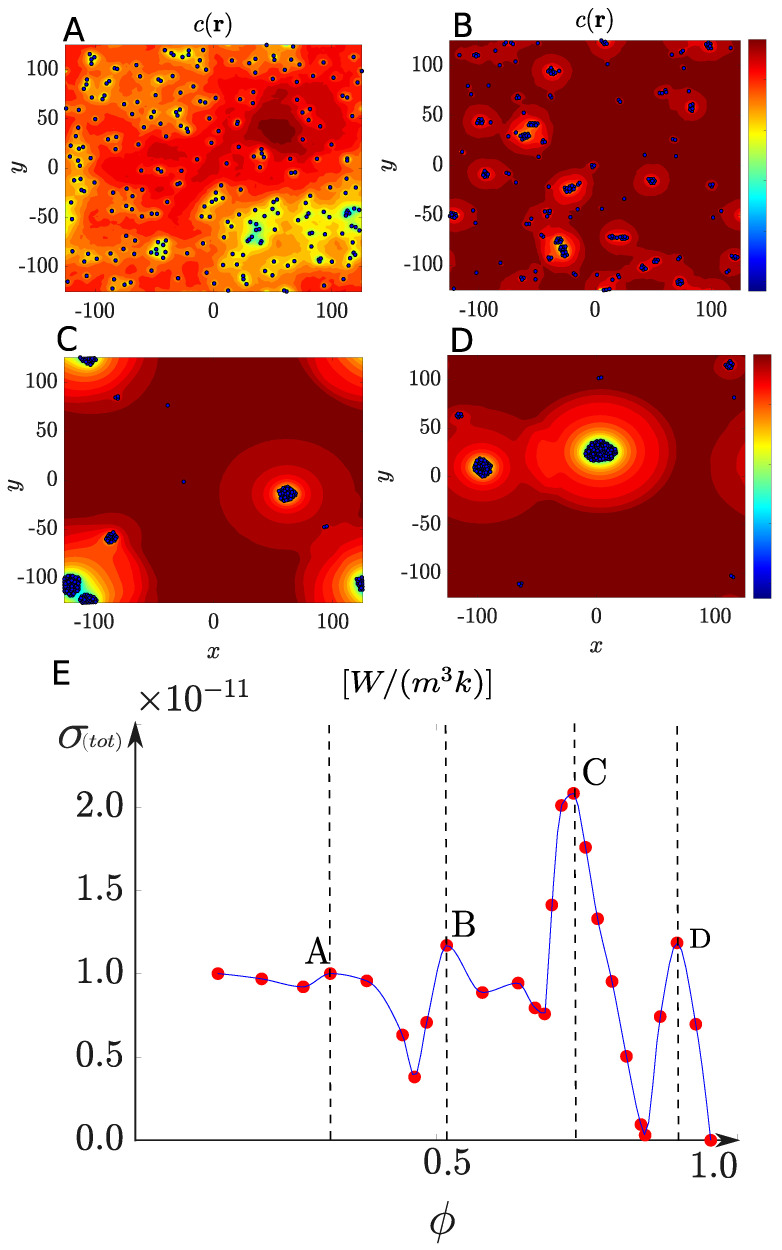
Structural regimes formed by active particles and their non-equilibrium thermodynamic characterization. The representations include both the structural types and the corresponding concentration fields for (**A**) active gas, (**B**) worm-like formations, (**C**) clustering, and (**D**) chemotactic collapse regimes. These results were obtained using Equations (2) and (3), with the c(r) profile obtained in [7], for different dimensionless values of $\xi_{t}$ and $\xi_{r}$. The parameters used were N=250, area fraction 2%, Pe=19, $\xi_{t}=\left[0,\;22\right]$, $\xi_{r}=\left[-1,\;2\right]$, particle radius of $1\times 10^{-6}$ m, and $D_{s}=1\times 10^{-11}\;m^{2}s^{-1}$. To avoid overlapping, a WCA potential was implemented [73]. (**E**) Entropy production computed from Equation (19), after solving particle dynamics, chemical reaction, and substrate diffusion equations, as a function of the structural parameter ϕ, for $T=T_{0}$, $T^{\left(B\right)}=T_{0}^{\left(B\right)}=T_{0}$, $C_{0}^{\left(B\right)}=0.1$ M, and $k_{r}=0.05\;s^{-1}$. The dashed lines indicate the transitions between distinct regimes.

By differentiating the entropy variations with respect to time and using the probability conservation law, we obtain the total entropy production rate in the system:

(16) $$\sigma_{\left(tot\right)}=-\frac{1}{T}\int J\left(\Gamma ,t\right)\cdot \nabla_{\Gamma }\mu \left(\Gamma ,t\right)d\Gamma$$

from which we can infer the probability current

(17) $$J(\Gamma ,t)=-\frac{L(\Gamma )}{k_{B}T}p\left(\Gamma ,t\right)\cdot \nabla_{\Gamma }\mu \left(\Gamma ,t\right)$$

with L(Γ) being an Onsager coefficient and $k_{B}$ the Boltzmann constant. Substituting the current in the probability conservation law leads to the Fokker–Planck equation:

(18) $$\frac{\partial p}{\partial t}=\nabla_{\Gamma }\cdot L\nabla_{\Gamma }p+\frac{Lp}{k_{B}T}\nabla_{\Gamma }\Psi$$

where we have used the expression for the chemical potential, $\mu =k_{B}T\ln p\left(\Gamma ,t\right)+\Psi \left(\Gamma \right)$, with Ψ being a generalized free-energy potential. From the Fokker–Planck equation (Equation (18)), we can obtain the Langevin equations (Equations (2) and (3)) describing the dynamics of the particles. By solving Equations (2) and (3), several structural regimes can be identified, as illustrated in Figure 4A–D. These regimes correspond to different values of the phoretic coefficients ($\xi_{t}$,$\xi_{r}$) raging between 0 and 22, and −1 and 2, respectively.

In Ref. [39], the MNET formalism was employed to derive Langevin and continuous equations consistent with the second law of thermodynamics, capturing the dynamics of catalytic Janus particles over time. In that framework, the total entropy production rate, $\sigma_{\left(tot\right)}$, is composed of two contributions: the interfacial entropy production, $\sigma_{i}$, evaluated at the *i*-th particle position and mainly given by the chemical reaction, and the bulk entropy production, $\sigma^{\left(B\right)}$, which is predominantly governed by substrate diffusion in bulk ($\nabla_{r}C_{s}^{\left(B\right)}$) and bulk variables ($D_{s}^{\left(B\right)},T_{0}^{\left(B\right)},C_{0}^{\left(B\right)},T^{\left(B\right)}$).

(19) $$\sigma_{\left(tot\right)}=\sum_{i=1}^{N}\sigma_{i}+\sigma^{\left(B\right)}\approx \frac{R_{g}T_{0}}{T}k_{r}\sum_{i=1}^{N}{\left(\Delta z\left(r_{i}\right)\right)}^{2}+\frac{D_{s}^{\left(B\right)}R_{g}T_{0}^{\left(B\right)}}{C_{0}^{\left(B\right)}T^{\left(B\right)}}{∥\nabla_{r}C_{s}^{\left(B\right)}∥}^{2}$$

When averaged over time, the mesoscopic entropy production provides a measure of dissipation (see Figure 4E). A variation in entropy as a function of the structural parameter, specifically the fraction of aggregated particles, is observed, highlighting the connection between system organization and thermodynamics.

A non-equilibrium free energy for active particles, $\mu_{AP}$, was also derived [39], as a function of the total entropy produced, $\Sigma =\int \int \sigma_{\left(tot\right)}drdt$, which provides fundamental insights into the thermodynamic feasibility of structure formation and the transition between different structures:

(20) $$N\Delta \mu_{AP}=-\frac{\partial H_{p}}{\partial \phi_{c}}+T\frac{\partial S_{p}}{\partial \phi_{c}}+T\frac{\partial \Sigma }{\partial \phi_{c}}$$

in which the free energy depends not only on particles enthalpy, $H_{p}$, and entropy, $S_{p}$, but also on the total entropy produced, Σ, derivative with respect to the fraction of assembled particles, $\phi_{c}$. This framework serves as an analogue to its equilibrium counterpart, extending free energy principles to systems operating far from equilibrium. Figure 4E is obtained from Equation (19), and it can be analyzed using Equation (20), as demonstrated in Ref. [39], to gain insights into regime transitions. Figure 4E was generated by calculating ϕ and $\sigma_{\left(tot\right)}$ for various combinations of $\xi_{t}$ and $\xi_{r}$.

Hydrodynamic effects significantly influence the collective dynamics of active particles, as revealed in recent studies [35]. Analyzing total entropy changes $\Delta S=\Delta_{r}S+\Sigma$, including reversible and irreversible contributions, as a function of hydrodynamic interaction strength, $k_{H}$, offers valuable insights into the most thermodynamically stable regime of active particles in a self-organized state resulting from the condition

(21) $$\frac{\partial \Delta S}{\partial k_{H}}=0$$

This relationship highlights how hydrodynamic effects influence the thermodynamic stability and organization of the system.

## 5. Discussions and Conclusions

This work emphasizes entropy production as a fundamental concept to understand not only the dynamics and thermodynamics of active particles but also to calculate phoretic and transport coefficients, as well as to characterize the collective structures that form and the different regimes in which they are encountered.

For single-particle dynamics, entropy production provides a rigorous framework for quantifying interfacial processes, elucidating the role of transport coefficients and the thermodynamic forces driving the motion, such as phoretic velocities. By capturing the interplay between temperature gradients, concentration, and surface properties, it offers a detailed characterization of the irreversible processes governing self-propulsion.

At the collective level, entropy production serves as a unifying metric to analyze regime transitions, structural organization, and the stability of emergent behaviors. From active gas formation to clustering to chemotactic collapse, it provides a consistent thermodynamic perspective on non-equilibrium phenomena. Recent theoretical advances in mesoscopic non-equilibrium thermodynamics bridge the gap between microscopic interactions and macroscopic behavior, enabling models that consistently describe active particle systems at all scales.

The integration of entropy production into the study of active particles reveals the underlying thermodynamic principles governing their behavior, offering a powerful tool for predicting regime transitions and characterizing structural phases. It also provides a basis for quantifying dissipation and energy conversion efficiency in these systems. Challenges remain, particularly in understanding the interplay between entropy production, concentration, temperature, and velocity fields in particle self-organization, as well as in manipulating transport coefficients to control particle dynamics. These open questions suggest interesting avenues for future research aimed at deepening our understanding of active matter.

## Data Availability

The original contributions presented in this study are included in the article. Further inquiries can be directed to the corresponding author.

## References

[1] Fu Y., Yu H., Zhang X., Malgaretti P., Kishore V., Wang W. (2022). Microscopic swarms: From active matter physics to biomedical and environmental applications. Micromachines.

[2] Gompper G., Winkler R.G., Speck T., Solon A., Nardini C., Peruani F., Löwen H., Golestanian R., Kaupp U.B., Alvarez L. (2020). The 2020 motile active matter roadmap. J. Phys. Condens. Matter.

[3] Kurzthaler C., Gentile L., Stone H.A. (2023). Out-of-Equilibrium Soft Matter.

[4] Liebchen B., Mukhopadhyay A.K. (2021). Interactions in active colloids. J. Phys. Condens. Matter.

[5] Mendelson N.H., Lega J. (1998). A Complex Pattern of Traveling Stripes Is Produced by Swimming Cells of Bacillus subtilis. J. Bacteriol..

[6] Bowick M.J., Fakhri N., Marchetti M.C., Ramaswamy S. (2022). Symmetry, thermodynamics, and topology in active matte. Phys. Rev. X.

[7] Pohl O., Stark H. (2014). Dynamic clustering and chemotactic collapse of self-phoretic active particles. Phys. Rev. Lett..

[8] Toschi F., Sega M. (2019). Flowing Matter.

[9] Fadda F., Matoz-Fernandez D.A., van Roij R., Jabbari-Farouji S. (2023). The interplay between chemo-phoretic interactions and crowding in active colloids. Soft Matter.

[10] Gaspard P., Kapral R. (2017). Communication: Mechanochemical fluctuation theorem and thermodynamics of self-phoretic motors. J. Chem. Phys..

[11] Gaspard P., Kapral R. (2018). Nonequilibrium thermodynamics and boundary conditions for reaction and transport in heterogeneous media. J. Chem. Phys..

[12] Prigogine I. (2017). Non-Equilibrium Statistical Mechanics.

[13] Derjaguin B., Sidorenkov G., Zubashchenkov E., Kiseleva E. (1947). Kinetic phenomena in boundary films of liquids. Kolloidn. Zh..

[14] Anderson J.L. (1989). Colloid Transport by Interfacial Forces. Annu. Rev. Fluid Mech..

[15] Bedeaux D., Albano A., Mazur P. (1976). Boundary conditions and non-equilibrium thermodynamics. Phys. Stat. Mech. Its Appl..

[16] Bafaluy J., Pagonabarraga I., Rubí J., Bedeaux D. (1995). Thermocapillary motion of a drop in a fluid under external gradients. Faxén theorem. Phys. Stat. Mech. Its Appl..

[17] Takatori S.C., Brady J.F. (2015). Towards a thermodynamics of active matter. Phys. Rev. E.

[18] Chaudhuri D. (2014). Active Brownian particles: Entropy production and fluctuation response. Phys. Rev. E.

[19] Burkholder E.W., Brady J.F. (2019). Fluctuation-dissipation in active matter. J. Chem. Phys..

[20] Speck T., Jack R.L. (2016). Ideal bulk pressure of active Brownian particles. Phys. Rev. E.

[21] Seifert U. (2019). From Stochastic Thermodynamics to Thermodynamic Inference. Annu. Rev. Condens. Matter Phys..

[22] Fodor E., Jack R.L., Cates M.E. (2022). Irreversibility and Biased Ensembles in Active Matter: Insights from Stochastic Thermodynamics. Annu. Rev. Condens. Matter Phys..

[23] Cates M.E., Fodor E., Markovich T., Nardini C., Tjhung E. (2022). Stochastic Hydrodynamics of Complex Fluids: Discretisation and Entropy Production. Entropy.

[24] Arango-Restrepo A., Rubi J.M., Barragán D. (2018). Understanding Gelation as a Nonequilibrium Self-Assembly Process. J. Phys. Chem..

[25] Reguera D., Rubi J.M., Vilar J.M.G. (2005). The mesoscopic dynamics of thermodynamic systems. J. Phys. Chem. B.

[26] De Groot S.R., Mazur P. (2013). Non-Equilibrium Thermodynamics.

[27] Rubi J., Bedeaux D., Kjelstrup S., Pagonabarraga I. (2013). Chemical cycle kinetics: Removing the limitation of linearity of a non-equilibrium thermodynamic description. Int. J. Thermophys..

[28] Olarte-Plata J.D., Bresme F. (2020). Orientation of Janus particles under thermal fields: The role of internal mass anisotropy. J. Chem. Phys..

[29] Huang M.J., Schofield J., Kapral R. (2017). Chemotactic and hydrodynamic effects on collective dynamics of self-diffusiophoretic Janus motors. New J. Phys..

[30] Gilbert T., Dorfman J.R. (1999). Entropy production: From open volume-preserving to dissipative systems. J. Stat. Phys..

[31] Vicsek T., Czirók A., Ben-Jacob E., Cohen I., Shochet O. (1995). Novel Type of Phase Transition in a System of Self-Driven Particles. Phys. Rev. Lett..

[32] Saha S., Golestanian R., Ramaswamy S. (2014). Clusters, asters, and collective oscillations in chemotactic colloids. Phy. Rev. E.

[33] Nasouri B., Golestanian R. (2020). Exact Phoretic Interaction of Two Chemically Active Particles. Phys. Rev. Lett..

[34] Scagliarini A., Pagonabarraga I. (2020). Unravelling the role of phoretic and hydrodynamic interactions in active colloidal suspensions. Soft Matter.

[35] Torrenegra-Rico J.D., Arango-Restrepo A., Rubi J.M. (2024). Self-organization of Janus particles: Impact of hydrodynamic interactions in substrate consumption for structure formation. J. Chem. Phys..

[36] Zhao H., Košmrlj A., Datta S.S. (2023). Chemotactic Motility-Induced Phase Separation. Phys. Rev. Lett..

[37] Ezhilan B., Pahlavan A.A., Saintillan D. (2012). Chaotic dynamics and oxygen transport in thin films of aerotactic bacteria. Phys. Fluids.

[38] Peng Z., Kapral R. (2024). Self-organization of active colloids mediated by chemical interactions. Soft Matter.

[39] Torrenegra-Rico J.D., Arango-Restrepo A., Rubí J.M. (2022). Nonequilibrium thermodynamics of Janus particle self-assembly. J. Chem. Phys..

[40] Bresme F., Olarte-Plata J.D., Chapman A., Albella P., Green C. (2022). Thermophoresis and thermal orientation of Janus nanoparticles in thermal fields. Eur. Phys. J..

[41] Jiang M., Chapman A., Olarte-Plata J.D., Bresme F. (2023). Controlling local thermal gradients at molecular scales with Janus nanoheaters. Nanoscale.

[42] Olarte-Plata J.D., Bresme F. (2024). Impact of the Interfacial Kapitza Resistance on Colloidal Thermophoresis. ACS Omega.

[43] Cao Z., Su J., Jiang H., Hou Z. (2022). Effective entropy production and thermodynamic uncertainty relation of active Brownian particles. Phys. Fluids.

[44] Ro S., Guo B., Shih A., Phan T.V., Austin R.H., Levine D., Chaikin P.M., Martiniani S. (2022). Model-Free Measurement of Local Entropy Production and Extractable Work in Active Matter. Phys. Rev. Lett..

[45] Dolai P., Maes C., Netočný K. (2023). Calorimetry for active systems. SciPost Phys..

[46] Paoluzzi M. (2022). Measuring Entropy in Active-Matter Systems. Physics.

[47] Onsager L. (1931). Reciprocal Relations in Irreversible Processes. II. Phys. Rev..

[48] Dufty J.W., Rub J.M. (1987). Generalized Onsager symmetry. Phys. Rev. A.

[49] Paxton W.F., Kistler K.C., Olmeda C.C., Sen A., St. Angelo S.K., Cao Y., Mallouk T.E., Lammert P.E., Crespi V.H. (2004). Catalytic Nanomotors:Autonomous Movement of Striped Nanorods. J. Am. Chem. Soc..

[50] Gaspard P., Kapral R. (2019). The stochastic motion of self-thermophoretic Janus particles. J. Stat. Mech. Theory Exp..

[51] Gaspard P., Kapral R. (2020). Active Matter, Microreversibility, and Thermodynamics. Research.

[52] Michelin S., Lauga E. (2014). Phoretic self-propulsion at finite Péclet numbers. J. Fluid Mech..

[53] Popescu M.N., Uspal W.E., Dietrich S. (2016). Self-diffusiophoresis of chemically active colloids. Eur. Phys. J. Spec. Top..

[54] Moran J.L., Posner J.D. (2017). Phoretic Self-Propulsion. Annu. Rev. Fluid Mech..

[55] De Corato M., Pagonabarraga I. (2022). Onsager reciprocal relations and chemo-mechanical coupling for chemically active colloids. J. Chem. Phys..

[56] Arango-Restrepo A., Rubi J.M. (2024). Interplay of phoresis and self-phoresis in active particles: Transport properties, phoretic, and self-phoretic coefficients. J. Chem. Phys..

[57] Pagonabarraga I., Pérez-Madrid A., Rubí J. (1997). Fluctuating hydrodynamics approach to chemical reactions. Phys. Stat. Mech. Its Appl..

[58] Martyushev L., Seleznev V. (2006). Maximum entropy production principle in physics, chemistry and biology. Phys. Rep..

[59] Arango-Restrepo A., Barragán D., Rubi J.M. (2019). Self-assembling outside equilibrium: Emergence of structures mediated by dissipation. Phys. Chem. Chem. Phys..

[60] Popescu M.N., Uspal W.E., Bechinger C., Fischer P. (2018). Chemotaxis of Active Janus Nanoparticles. Nano Lett..

[61] Klamser J.U., Kapfer S.C., Krauth W. (2018). Thermodynamic phases in two-dimensional active matter. Nat. Commun..

[62] Cates M.E., Tailleur J. (2015). Motility-induced phase separation. Annu. Rev. Condens. Matter Phys..

[63] Stenhammar J., Marenduzzo D., Allen R.J., Cates M.E. (2014). Phase behaviour of active Brownian particles: The role of dimensionality. Soft Matter.

[64] Takatori S.C., Brady J.F. (2016). Forces, stresses and the (thermo?) dynamics of active matter. Curr. Opin. Colloid Interface Sci..

[65] Caprini L., Marini Bettolo Marconi U., Puglisi A. (2019). Activity induced delocalization and freezing in self-propelled systems. Sci. Rep..

[66] Zhang Y., Barato A.C. (2016). Critical behavior of entropy production and learning rate: Ising model with an oscillating field. J. Stat. Mech. Theory Exp..

[67] Seara D.S., Yadav V., Linsmeier I., Tabatabai A.P., Oakes P.W., Tabei S.M.A., Banerjee S., Murrell M.P. (2018). Entropy production rate is maximized in non-contractile actomyosin. Nat. Commun..

[68] Noa C.E.F., Harunari P.E., de Oliveira M.J., Fiore C.E. (2019). Entropy production as a tool for characterizing nonequilibrium phase transitions. Phys. Rev. E.

[69] Shklyaev O.E., Shum H., Balazs A.C. (2018). Using Chemical Pumps and Motors To Design Flows for Directed Particle Assembly. Accounts Chem. Res..

[70] O’Byrne J., Solon A., Tailleur J., Zhao Y. (2023). An Introduction to Motility-induced Phase Separation. Out-of-Equilibrium Soft Matter: Active fluids.

[71] Mandal D., Klymko K., DeWeese M.R. (2017). Entropy Production and Fluctuation Theorems for Active Matter. Phys. Rev. Lett..

[72] Arango-Restrepo A., Barragán D., Rubi J.M. (2021). A Criterion for the Formation of Nonequilibrium Self-Assembled Structures. J. Phys. Chem..

[73] Weeks J.D., Chandler D., Andersen H.C. (1971). Role of repulsive forces in determining the equilibrium structure of simple liquids. J. Chem. Phy..

